# Gender Differences in the Amount and Type of Student Participation During In-Person and Virtual Classes in Academic Medicine Learning Environments

**DOI:** 10.1001/jamanetworkopen.2021.43139

**Published:** 2022-01-12

**Authors:** Sara J. Cromer, Kristin M. D’Silva, Neelam A. Phadke, Emma Lord, Nancy A. Rigotti, Heather J. Baer

**Affiliations:** 1 Division of Endocrinology, Diabetes, and Metabolism, Massachusetts General Hospital, Boston; 2 Harvard Medical School, Boston, Massachusetts; 3 Division of Rheumatology, Allergy, and Immunology, Massachusetts General Hospital, Boston; 4 Northeastern University, Boston, Massachusetts; 5 Division of General Internal Medicine, Massachusetts General Hospital, Boston; 6 Division of General Medicine and Primary Care, Brigham and Women’s Hospital, Boston, Massachusetts; 7 Department of Epidemiology, Harvard T.H. Chan School of Public Health, Boston, Massachusetts

## Abstract

This cohort study examines gender differences in the amount and type of student participation during in-person and virtual classes in graduate-level academic medicine learning environments.

## Introduction

Despite the increased representation of women in medicine, disparities persist in how women and men assert and promote themselves in academic settings. We examined gender differences in the amount and type of student participation during in-person and virtual classes in graduate-level academic medicine learning environments.

## Methods

This cohort study was approved by the Mass General Brigham Institutional Review Board. Full informed consent was waived in accordance with the Federal Policy for the Protection of Human Subjects; however, all students were aware of observation and data collection, and students in 2020 (when individual-level data were collected) were given the opportunity to opt out of the study. The study followed the Strengthening the Reporting of Observational Studies in Epidemiology (STROBE) reporting guideline.

Students in a graduate-level certificate program were observed during large, lecture-based classes and smaller, discussion-based classes for 2 weeks in July 2019 (in-person) and 6 weeks from July to August 2020 (virtual).

The primary study outcome was the number of questions asked. Secondary outcomes included the number of questions answered and the use of predefined deferential language (eTable in the Supplement) when asking questions. Incidence rate ratios (IRRs) were calculated as the number of questions or answers per woman-hour vs man-hour of observation. The relative risk (RR) of a question containing deferential language was calculated using the number of deferential questions and nondeferential questions among each gender. A 2-sided *P* value of <.05 was considered statistically significant. Wald 95% CIs were calculated for all point estimates, and a Fisher exact test was used for comparisons between class sizes and years. Analyses were stratified by class size and setting and were conducted using R, version 4.0.2 (R Core Team, 2020).

## Results

In 2019, 156 students were observed; 94 (60%) were women and 147 (94%) were physicians. In 2020, 138 students were observed; 84 (61%) were women and 130 (94%) were physicians. During 2019 in-person classes (Table 1), women had a lower rate of asking (IRR, 0.44 [95% CI, 0.36-0.55]) and answering (IRR, 0.28 [95% CI, 0.19-0.39]) questions than men in large but not small classes. During 2020 large virtual classes, women had a similar rate of asking questions but had a lower rate of answering questions (IRR, 0.67 [95% CI, 0.57-0.80]) than men; in small classes, women had a higher rate of asking questions than men (IRR, 1.86 [95% CI, 1.08-3.32]), with a similar rate of answering questions. Deferential language was used more frequently in questions asked by women than men in large classes (2019 in-person classes: RR, 1.48 [95% CI, 1.00-2.18]; and 2020 virtual classes: RR, 2.03 [95% CI, 1.49-2.76]) but not in small classes.

**Table 1.** Baseline Characteristics and Aggregate Speech Outcomes by Gender in Small and Large Classes, 2019 and 2020

	2019 in-person classes				2020 virtual classes			
	Aggregate counts, No. (%)		Women vs men, effect size (95% CI)^a^		Aggregate counts, No. (%)		Women vs men, effect size (95% CI)^a^	
	Large (n = 156)	Small (n = 31)	Large (n = 156)	Small (n = 31)	Large (n = 138)	Small (n = 31)	Large (n = 138)	Small (n = 31)
**Participant characteristics**								
Sex								
Women	94 (60)	22 (71)	NA	NA	84 (61)	19 (61)	NA	NA
Men	62 (40)	9 (29)	NA	NA	54 (39)	12 (39)	NA	NA
MD or DO degree	147 (94)	NA	NA	NA	130 (94)	31 (100)	NA	NA
Stage of training: fellowship	104 (67)	NA	NA	NA	98 (71)	26 (84)	NA	NA
Country of residence: US	123 (79)	NA	NA	NA	115 (83)	29 (94)	NA	NA
**Speech behaviors**								
Person-hours observed								
Women	2119.5	330	NA	NA	4903	389	NA	NA
Men	1225.5	135	NA	NA	3579	254	NA	NA
Questions asked (aloud)								
Women^b^	155	41	IRR, 0.44 (0.36-0.55)	IRR, 0.76 (0.44-1.34)	270	54	0.99 (0.82-1.20)	1.86 (1.08-3.32)
Men	202	22	1 [Reference]	1 [Reference]	199	19	1 [Reference]	1 [Reference]
Questions answered (aloud)								
Women^b^	49	140	IRR, 0.28 (0.19-0.39)	IRR, 1.02 (0.75-1.42)	264	59	0.67 (0.57-0.80)	1.07 (0.70-1.67)
Men	102	56	1 [Reference]	1 [Reference]	286	36	1 [Reference]	1 [Reference]
Deferential language (aloud)								
Women^c^	42	10	RR, 1.48 (1.00-2.18)	RR, 0.67 (0.31-1.45)	113	11	2.03 (1.49-2.76)	0.65 (0.28-1.50)
Men	37	8	1 [Reference]	1 [Reference]	41	6	1 [Reference]	1 [Reference]

Abbreviations: IRR, incidence rate ratio; NA, not available; RR, relative risk.

^a^ Wald 95% CIs.

^b^ IRRs (for total questions asked or answered) were calculated using aggregate numbers of questions or answers, respectively, and total person-hours of observation time.

^c^ RRs (for deferential language) were calculated using aggregate deferential language and aggregate numbers of questions for women vs men.

In analyses by class size (Table 2), questions were less frequently asked (2019 in-person classes: RR, 0.67 [95% CI, 0.54-0.83]; and 2020 virtual classes: RR, 0.78 [95% CI, 0.67-0.91]) and answered (2019 in-person classes: RR, 0.45 [95% CI, 0.36-0.58]; and 2020 virtual classes: RR, 0.77 [95% CI, 0.65-0.92]) by women in large compared with small classes. During 2020 virtual classes, questions asked by women more frequently contained deferential language in large compared with small classes (RR, 2.05 [95% CI, 1.19-3.55]), with no difference during 2019 in-person classes. During 2019 in-person classes, questions from men less frequently contained deferential language in large compared with small classes (RR, 0.50 [95% CI, 0.27-0.94]), with no difference during 2020 virtual classes.

**Table 2.** Comparison of Speech Behaviors in Large vs Small Classes and in 2019 In-Person vs 2020 Virtual Classes

Exposure (class size or setting)	Speech behavior outcome	RR (95% CI)
Large vs small classes, stratified by class setting		
2019 in-person	Questions asked by women^a^	0.67 (0.54-0.83)
	Questions answered by women	0.45 (0.36-0.58)
	Use of deferential language in questions from women^b^	1.11 (0.61-2.02)
	Use of deferential language in questions from men	0.50 (0.27-0.94)
2020 virtual	Questions asked by women^a^	0.78 (0.67-0.91)
	Questions answered by women	0.77 (0.65-0.92)
	Use of deferential language in questions from women	2.05 (1.19-3.55)
	Use of deferential language in questions from men	0.65 (0.32-1.33)
2019 in-person vs 2020 virtual classes, stratified by class size		
Large	Questions asked by women^a^	0.75 (0.65-0.87)
	Questions answered by women	0.68 (0.53-0.85)
	Use of deferential language in questions from women^b^	0.65 (0.48-0.87)
	Use of deferential language in questions from men	0.89 (0.60-1.32)
Small	Questions asked by women^a^	0.88 (0.70-1.10)
	Questions answered by women	1.15 (0.96-1.38)
	Use of deferential language in questions from women^b^	1.20 (0.56-2.54)
	Use of deferential language in questions from men	1.15 (0.49-2.73)

Abbreviation: RR, relative risk.

^a^ RR of questions being asked or answered by a woman (among all questions asked or answered) vs by a man in (1) large vs small classes and (2) 2019 in-person vs 2020 virtual classes.

^b^ RR of questions containing deferential language if asked by women or men (among all questions asked by that gender) in (1) large vs small classes and (2) 2019 in-person vs 2020 virtual classes.

In analyses by class setting (Table 2), questions were less frequently asked (RR, 0.75 [95% CI, 0.65-0.87]) and answered (RR, 0.68 [95% CI, 0.53-0.85]) by women during in-person compared with virtual classes in large but not in small classes. Questions asked by women less frequently contained deferential language during in-person compared with virtual classes in large classes (RR, 0.65 [95% CI, 0.48-0.87]), with no differences by class setting for men in large classes or either gender in small classes.

## Discussion

In this cohort study, women had a lower rate of asking and answering questions and were more likely to use deferential language in large classes than men, but these disparities were attenuated in smaller, discussion-based and virtual classes. These findings are consistent with studies demonstrating that women assert themselves differently from men in academic settings,^1,2,3,4^ although these differences are attenuated in smaller classes.^2,5^ These differences have been associated with less positive evaluations and lower rates of promotion,^2,3,6^ suggesting that systems of evaluation may be biased toward characteristics more common in men.

The limitations of this study include performance at a single institution, aggregate rather than individual-level data analysis, and potential for confounding. Because gendered differences in speech behaviors have been associated with academic advancement, it is critical to reexamine methods of physician evaluation and promotion to avoid systemic bias.

## Notes

Supplement. **eTable.** Examples of Deferential Language
