# Supplementary material for: Gender Differences in the Amount and Type of Student Participation During In-Person and Virtual Classes in Academic Medicine Learning Environments
Source: JAMA Netw Open. 2022 Jan 12;5(1):e2143139. doi: 10.1001/jamanetworkopen.2021.43139 (PMC8756329; doi:10.1001/jamanetworkopen.2021.43139)
Supplement: Supplement. — eTable. Examples of Deferential Language [file jamanetwopen-e2143139-s001.pdf]

Supplementary Online Content

Cromer SJ, D'Silva KM, Phadke NA, Lord E, Rigotti NA, Baer HJ. Gender differences in the amount and type of student participation during in-person and virtual classes in academic medicine learning environments. *JAMA Netw Open*. 2022;5(1):e2143139. doi:10.1001/jamanetworkopen.2021.43139

eTable. Examples of Deferential Language

This supplementary material has been provided by the authors to give readers additional information about their work.

© 2022 Cromer SJ et al. *JAMA Network Open*.

eTable. Examples of Deferential Language

Category of Deferential Language	Examples
Requesting permission	"Could I ask a question?"<br>"Can [May] I ask a quick question?"<br>"Can I ask something?"<br>"Can I clarify?"
Apologetic	"Sorry, quick question…"<br>"Sorry, I probably missed this part…"<br>"Sorry to bother you…"<br>"Sorry, last question."
Diminishing the speaker or question	"I think where I'm having trouble with what you're describing is…"<br>"This is going to be a silly question."<br>"I have a very small understanding of this, but…"<br>"I have a silly example."<br>"Hopefully I'm phrasing this right…"<br>"Maybe I'm not understanding correctly."<br>"I could be wrong…"<br>"I hope this isn't a stupid question…"
"Just" language	"I just have a question…"<br>"Just to clarify…"<br>"I was just going to add…"<br>"That's just my guess"<br>"I was just wondering…"<br>"I was just hoping…"
Other or mixed deferential phrasing	"You may get to this, but…"<br>"I'm just wondering, or I'm still a little bit confused about…"<br>"This may be off-topic, but…"<br>"I just have a really simple question because I'm struggling for some reason."<br>"I don't know if it's me or if others feel this way, but…"<br>"I'm sorry – this is a weird question; I guess I don't understand why…"<br>"So, just on that last slide, maybe I'm dumbing it down a bit…"<br>"I think I just struggle a lot with…"<br>"I just want to make sure I'm understanding this correctly"<br>"Can I ask just one quick question?"

© 2022 Cromer SJ et al. *JAMA Network Open*.
